# Sleep and Loneliness as Mechanisms Through Which Marital Quality Predicts Depression Among Older Adults

**DOI:** 10.1093/geroni/igab046.127

**Published:** 2021-12-17

**Authors:** Christina Marini, Lynn Martire, Orfeu Buxton

**Affiliations:** 1 Adelphi University, Garden City, New York, United States; 2 The Pennsylvania State University, University Park, Pennsylvania, United States; 3 The Pennsylvania State University, The Pennsylvania State University, Pennsylvania, United States

## Abstract

Pathways through which spousal support and strain influence older adults’ well-being are poorly understood. We examined sleep quality and loneliness as mechanisms through which support and strain predict depressive symptoms across ten years utilizing National Social Life, Health, and Aging Project data. Our sample included partnered participants at waves 1 and 2 (N = 1,293; 39% female, M age = 66, SD = 6.93). Support (e.g., rely on spouse) and strain (e.g., spouse criticizes you) were measured at W1, loneliness (UCLA) and sleep quality (restless sleep) were measured at W2, and depression (CES-D) was measured at W3. We estimated latent-variable structural equation models, controlling for age, gender, and W1 depression. Indirect effects of support and strain on depressive symptoms through loneliness were significant. There was an additional trend-level indirect effect of spousal strain on depressive symptoms through restless sleep. Findings highlight multiple pathways through which marital quality predicts later-life well-being.

